# A Mutant Era GTPase Suppresses Phenotypes Caused by Loss of Highly Conserved YbeY Protein in *Escherichia coli*

**DOI:** 10.3389/fmicb.2022.896075

**Published:** 2022-05-19

**Authors:** Vignesh M. P. Babu, Siva Sankari, Anubrata Ghosal, Graham C. Walker

**Affiliations:** Department of Biology, Massachusetts Institute of Technology, Cambridge, MA, United States

**Keywords:** YbeY, Era, endoribonuclease, GTPase, ribosome assembly, rRNA processing

## Abstract

Ribosome assembly is a complex fundamental cellular process that involves assembling multiple ribosomal proteins and several ribosomal RNA species in a highly coordinated yet flexible and resilient manner. The highly conserved YbeY protein is a single-strand specific endoribonuclease, important for ribosome assembly, 16S rRNA processing, and ribosome quality control. In *Escherichia coli, ybeY* deletion results in pleiotropic phenotypes including slow growth, temperature sensitivity, accumulation of precursors of 16S rRNA, and impaired formation of fully assembled 70S subunits. Era, an essential highly conserved GTPase protein, interacts with many ribosomal proteins, and its depletion results in ribosome assembly defects. YbeY has been shown to interact with Era together with ribosomal protein S11. In this study, we have analyzed a suppressor mutation, *era(T99I)*, that can partially suppress a subset of the multiple phenotypes of *ybeY* deletion. The *era(T99I)* allele was able to improve 16S rRNA processing and ribosome assembly at 37°C. However, it failed to suppress the temperature sensitivity and did not improve 16S rRNA stability. The *era(T99I)* allele was also unable to improve the 16S rRNA processing defects caused by the loss of ribosome maturation factors. We also show that *era(T99I)* increases the GroEL levels in the 30S ribosome fractions independent of YbeY. We propose that the mechanism of suppression is that the changes in Era’s structure caused by the *era(T99I)* mutation affect its GTP/GDP cycle in a way that increases the half-life of RNA binding to Era, thereby facilitating alternative processing of the 16S RNA precursor. Taken together, this study offers insights into the role of Era and YbeY in ribosome assembly and 16S rRNA processing events.

## Introduction

YbeY protein is a highly conserved protein found in most bacteria and also in the eukaryotic organelles of bacterial origin, including mitochondria and chloroplasts (Liao et al., 2021). YbeY was identified as a protein encoded by a gene that was found to be essential for symbiosis between the alfalfa plant and *Sinorhizobium meliloti* bacteria (Davies and Walker, 2008). The subsequent characterization of YbeY in *Escherichia coli* showed it to be a heat shock protein that is involved in the process of translation (Rasouly et al., 2009, 2010). *ybeY* deletion leads to severe growth defects and ribosome assembly defects that result in an accumulation of both the small 30S and the large 50S ribosomal subunits and in reduced levels of mature 70S ribosomes (Rasouly et al., 2009; Davies et al., 2010). Although structural studies of YbeY initially suggested that it is a metal-dependent protease or hydrolase, no protease activity was detected (Oganesyan et al., 2003; Yeh et al., 2004; Hervé Du Penhoat et al., 2005; Zhan et al., 2005). However, YbeY was then shown to possess a single-strand specific endoribonuclease activity (Jacob et al., 2013) that is zinc-dependent (Babu et al., 2019). The absence of YbeY also causes 16S rRNA processing defects that lead to the accumulation of 17S rRNA precursors (Davies et al., 2010). In addition, 16S rRNA degradation products (16S*) are present in these cells, suggesting that YbeY plays a role in rRNA stability. RNase R and YbeY were shown to act together in a ribosome quality control process that eliminates defective ribosomes (Jacob et al., 2013). YbeY is required for the virulence of some pathogenic bacteria namely *Pseudomonas aeruginosa* (Xia et al., 2020), *Vibrio cholerae* (Vercruysse et al., 2014), enterohemorrhagic *E. coli* (McAteer et al., 2018), and *Brucella abortus* (Budnick et al., 2018). Interestingly, YbeY has also been shown to be important for bacterial longevity (Pechter et al., 2017).

YbeY’s single-strand endonuclease activity has only limited intrinsic specificity, but several pieces of evidence suggest that YbeY can be guided to play its highly specific physiological roles through interactions with other proteins. Of particular interest, YbeY has been shown to interact with ribosomal proteins including the Era, S11, and Der (Vercruysse et al., 2016). Era is an essential protein and is one of the multiple ribosome-associated GTPases (RA-GTPases). In addition to Era, RA-GTPases such as RsgA, Der, BipA, and ObgE function in various stages of the ribosome assembly process (Britton, 2009; Gibbs et al., 2020; Goh et al., 2021). Era binds near the 3′ end of the 16S rRNA of the 30S subunit (Sayed et al., 1999; Tu et al., 2009, 2011). Human YbeY (C21orf57) could partially complement defects due to loss of YbeY in *E. coli* (Ghosal et al., 2017) and is also shown to be required for tRNA processing in human mitochondria (D’Souza et al., 2021). The interaction between YbeY, S11, and Era and the ribosome assembly function of YbeY is conserved in human mitochondria (Summer et al., 2020). Era depletion results in ribosome assembly defects and accumulation of defective ribosomal particles (Razi et al., 2019). We previously showed that overexpression of Era can partially suppress phenotypes due to a lack of YbeY (Ghosal et al., 2018). This close functional relationship between YbeY, S11, and Era together with YbeY’s endoribonuclease activity suggests a direct role for YbeY in 16S rRNA processing. However, a direct *in vitro* demonstration of 16S maturation by YbeY is still lacking. It remains to be determined if the pleiotropic phenotypes of a Δ*ybeY* mutant originate from a single functional defect or if YbeY has multiple independent functions. Identification of separation of function mutations might help answer these questions. The severe growth defect due to lack of YbeY allows for the easy identification of larger colonies that might possess one or more suppressors which might compensate for one or more of YbeY phenotypes.

In this study, we performed whole-genome sequencing of *ybeY* deletion strains with improved growth characteristics and have characterized a point mutation in the *era* gene, *era(T99I)*, that encodes an Era protein variant whose threonine residue at position 99 is replaced by an Isoleucine residue. We have determined that *era(T99I)* is responsible for the suppression of a subset of the phenotypes of a Δ*ybeY* mutant but not the others. We show that *era(T99I)* improves the slow growth, 16S rRNA processing defect, and ribosome assembly defect of the *ybeY* deletion strain but not its temperature sensitivity and 16S rRNA stability. We also show that the phenotypic suppression by *era(T99I)* is not dependent on a single exoribonuclease. The suppression is specific to the 16S rRNA processing defect due to *ybeY* deletion and does not improve the 16S rRNA defects due to the loss of maturation factors like KsgA, RimM, RsgA, and RbfA. Furthermore, we show that the presence of *era(T99I)* allele results in the accumulation of the chaperone GroEL in the 30S ribosome fractions of both *ybeY*+ and Δ*ybeY*. Our analysis suggests that the *era(T99I)* mutation could result in structural changes affecting the GTP/GDP binding to Era thereby affecting its half-life on the 16S rRNA precursors and enabling proper processing and ribosome assembly.

## Materials and Methods

### Bacterial Strains and Growth Conditions

*Escherichia coli* strain MC4100 was used as the wild-type strain throughout this study. Strains, plasmids, and primers used in this study are listed in Supplementary Table 1. Standard LB media was used throughout the study to grow the strains. Kanamycin (40 μg/ml), chloramphenicol (20 μg/ml), and tetracycline (10 μg/ml) were used whenever strains carrying the resistance markers were grown.

### Isolation of Δ*ybeY(sup1)* Strain

The *ybeY* knockout strain (JW0656) was used as the source of the Kanamycin resistance gene at the *ybeY* locus. P1(vir) transduction (Sambrook and Russell, 2001) was used to move the Kanamycin cassette into wild-type MC4100. The Kanamycin resistance gene at the YbeY locus was then removed using a temperature-sensitive plasmid pCP20 (Cam^R^) at 30°C. The pCP20 plasmid was cured by re-streaking the colonies at 37°C followed by screening for the absence of both chloramphenicol and kanamycin. Some colonies with relatively larger colony size appear during this process and were further examined. One such colony named the Δ*ybeY(sup1)* strain is characterized in this study.

### Generating Clean YbeY Deletion Strains

We utilized the λ Red recombination system (pKM208, Amp^R^) to introduce the kanamycin resistance gene into the YbeY gene locus using primers FP_*ybeY*_KO and RP_*ybeY*_KO to create a YbeY knockout strain. The pKM208 plasmid is then cured by re-streaking at 37°C to generate the Δ*ybeY::Kan(donor)* strain. This Δ*ybeY::Kan(donor)* strain is then utilized as the P1 donor for the Kanamycin cassette that knocks out *ybeY* in the MC4100 strain. The colonies picked from the transduction plates were grown in liquid LB once and frozen as glycerol stocks at −80°C. By this method, Δ*ybeY::Kan* strain used in this study was created without utilizing the Keio knockout collection and thus avoids the carryover of any linked mutations present in the Keio collection strain. All strains carrying Δ*ybeY* allele were made by knocking out *ybeY* gene as the last step (Supplementary Figure 1).

### Isolating *era(T99I)* Mutation From Δ*ybeY(sup1)*

The *era(T99I)* mutation present in the Δ*ybeY(sup1)* strain was moved into the MC4100 [*ybeY(*+)] strain by P1vir transduction. The steps involved are summarized in Supplementary Figure 2. First, we amplified the *tetA-sacB* cassette from the XTL298 strain using primers rncuptetAFor and rncupSacBRev and then utilized the λ Red recombination system to introduce a *tetA-sacB* cassette upstream of the *rnc* gene promoter (Supplementary Table 1). Attempts were made to transduce the era mutant gene from the Δ*ybeY(sup1)* strain followed by selection with Fusaric acid and sucrose as described previously (Li et al., 2013). This method was unsuccessful in the transfer of era mutation. We, therefore, transduced *glyA::Kan* allele from Keio collection into the *tetA-sacB* strain to generate a strain that carries the Kanamycin resistance gene on one side of the *era* gene and the tetracycline resistance gene on the other side of the *era* gene. In addition to resistance to kanamycin and tetracycline, this strain is also unable to grow on minimal media without amino acids. The *era* mutant allele was then transduced from the Δ*ybeY(sup1)* strain into the *glyA::Kan tetA-sacB* strain and selected on M9 minimal media without amino acids. The colonies are then screened for the absence of tetracycline and kanamycin. All colonies that satisfied these criteria were confirmed to have *era(T99I)* mutation by Sanger sequencing.

### rRNA Analysis, Ribosomal Fractionation, and Mass Spectrometry

For ribosomal RNA analysis, total RNA was extracted from the frozen cell pellets collected from the early exponential phase (OD_600nm_ ∼0.3) using TRIzol reagent (Thermo Fisher Scientific, Waltham, MA, United States) and RNeasy mini kit (Qiagen, Beverly, MA, United States). The total RNA is then mixed 1:1 with the RNA loading dye (NEB) and run on a Synergel-Agarose gel (1.6%). The separation of ribosomal particles was performed using the sucrose gradient method described previously (Ghosal et al., 2018). Briefly, 500 ml cultures at exponential phase (OD_600nm_ ∼0.3) were pelleted, resuspended in 5 ml Buffer A [20 mM HEPES pH 7.5, 5 mM β-mercaptoethanol, 10 mM MgCl_2_, 50 mM NH_4_Cl, and protease inhibitor cocktail (Roche)] and lysed using a French press. 200 μl of the lysates were then loaded onto 10 ml 10–40% sucrose gradients. The gradients were then ultra-centrifuged in an SW41 rotor (Beckman Coulter) at 150,000 *g* for 16 h with slow deceleration. 250 μl fractions were separated manually into 96-well plates followed by OD_260nm_ measurements.

The ribosomal fractions were run on SDS-PAGE gel followed by in-gel trypsin digestion. The samples were then analyzed by chromatographic separation followed by Mass spectrometry (Thermo Fisher Scientific Orbitrap Elite). The spectrum counts obtained were filtered (using Scaffold software) with the parameters such as peptide threshold of 20%, minimum peptide count of 2, and protein threshold of 20%. The spectrum counts of each subunit fractions were then normalized by dividing the raw spectrum count of a protein to the sum of all the spectrum counts in that fraction. The relative spectrum counts thus obtained were then plotted and the proteins whose counts differ by more than 20% were considered for further analysis.

### Whole Genome Sequencing and Analysis

Genomic DNA was extracted from overnight cultures using the GenElute Bacterial Genomic DNA kit (Sigma) as per the manufacturer’s instructions. Sequencing libraries were generated using Nextera XT DNA Library Prep Kit (NEB). Single-end sequencing was then performed using MiSeq: 50 nt v2 kit (Illumina). The reads obtained were then analyzed using the galaxy server^1^ (Afgan et al., 2018). The fastq reads were first aligned to reference MC4100 wild-type genome (GenBank HG738867.1) using the Bowtie2 tool (Langmead and Salzberg, 2012). The BAM alignments were subjected to variant analysis to identify mutations using the Naïve Variant Call tool (Blankenberg et al., 2014). Variants were then annotated using the SnpEff eff tool (Cingolani et al., 2012). Variant calls that have an allelic frequency of 1 and allele count or read depth of at least 5 were chosen as confident calls. The calls were further filtered for strand biases and the mutations identified were then verified by Sanger sequencing.

## Results

### Characterization of a Δ*ybeY* Derivative With an Improved Growth Phenotype

To make fresh derivatives of *E. coli* strain MC4100 that lacked *ybeY*, we had frequently used P1 phage to transduce the *ybeY* knockout allele carrying a kanamycin resistance gene from the Keio Collection strain JW0656 into the recipient wild type MC4100 strain. The kanamycin resistance gene at Δ*ybeY* locus was then removed introducing the Flp recombinase expressing plasmid, which was subsequently cured by temperature shifts and screening for loss of antibiotic resistance (see section “Materials and Methods”). After this multiple step process, we noticed occasional MC4100 Δ*ybeY* colonies with larger colony size and became interested in determining the molecular basis of the colony size increase. In this study, we report our characterization of one such isolate, provisionally designated as strain Δ*ybeY(sup1)*, that had striking and consistent phenotypes. We were initially unsure as to whether that the multiple steps involved in the YbeY deletion generation process might have favored the acquisition of a spontaneously arising suppressor or alternatively whether there could have been a genetically linked suppressor in Keio Collection strain JW0656 that was sometimes transferred as well by co-transduction with the *ybeY* allele. We therefore employed λ Red recombination to generate a new Δ*ybeY* allele marked by a kanamycin resistance gene (Δ*ybeY::Kan*) that did not come from the Keio collection (see section “Materials and Methods” and Supplementary Figure 1). A fresh MC4100 derivative carrying this allele (represented as Δ*ybeY::Kan*) was constructed by P1 transduction and is used throughout this study as the *ybeY* deletion control. The Kanamycin cassette was not removed to avoid further manipulation steps. The parental MC4100 strain is represented as *ybeY(*+) and used as the wild-type control.

**FIGURE 1:**
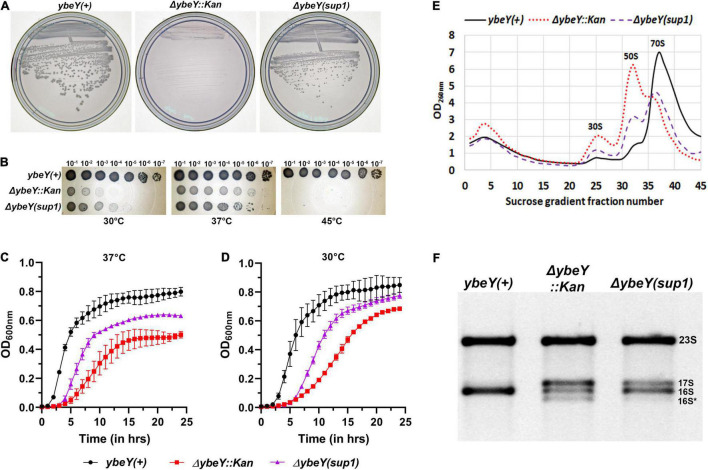
Characterization of the suppressed Δ*ybeY* isolate. **(A)** Strains from freezer stocks were streaked onto LB agar plates and then incubated at 37°C overnight. Plates were imaged separately. An increase in colony formation and colony diameter was observed in the Δ*ybeY(sup1)* strain compared to the Δ*ybeY::Kan* strain. **(B)** Tenfold serial dilutions of the overnight cultures were spotted onto LB agar plates and incubated at the indicated temperatures overnight. Representative sections of the imaged plates are shown. **(C,D)** Growth curves of the strains *ybeY*+ (black, circles), Δ*ybeY(sup1)* (purple, triangles), and Δ*ybeY::Kan* (red, squares) in liquid LB media were determined at 37°C **(C)** or 30°C **(D)** by measuring optical density (OD) at 600 nm in a plate reader for 24 h. The average ± SD of three determinations is shown. **(E)** Total cell lysate of the strains *ybeY*+ (black, continuous line), Δ*ybeY(sup1)* (purple, broken line), and Δ*ybeY::Kan* (red, dotted line), separated by sucrose gradients and the amount of RNA in each fraction was determined by measuring absorbance at 260 nm. **(F)** Total RNA samples were separated on a Synergel-agarose gel by electrophoresis. Section of the representative gel image with 23S and 16S bands is shown.

When these three strains were streaked from frozen stocks onto LB agar and incubated at 37°C overnight, we noted that the Δ*ybeY::Kan* strain formed strikingly smaller colonies than the *ybeY(*+) strain, an observation we have previously reported (Davies et al., 2010; Figure 1A). In contrast, the Δ*ybeY(sup1)* strain grew noticeably faster than the Δ*ybeY::Kan* strain and formed larger colonies. To examine the effect of growth temperature, the three strains were inoculated into liquid LB media and grown at 37°C overnight. These cultures were serially diluted and spotted on LB agar plates, which were then incubated at 30, 37, or 45°C. At 45°C, none of the strains lacking *ybeY* were able to grow. At 37°C, the Δ*ybeY::Kan* and Δ*ybeY(sup1)* strains formed visible growth spots at the same dilutions, although the colonies within the spots of the Δ*ybeY(sup1)* strain were relatively larger as expected (Figure 1B). At 30°C, both the strains lacking *ybeY* grew slower compared to *ybeY(*+), but the Δ*ybeY(sup1)* strain had more robust spot growth compared to the Δ*ybeY::Kan* strain. This result indicates that, while the suppressor present in the Δ*ybeY(sup1)* strain improves growth at 30 and 37°C, it does not suppress the temperature sensitivity of the Δ*ybeY::Kan* strain.

To determine the extent of suppression of the growth phenotype, growth curves were determined in the liquid LB media. At 37°C, Δ*ybeY::Kan* strain grew significantly slower (doubling time of 57.4 ± 8.3 min during exponential phase) compared to the *ybeY(*+) strain (doubling time of 23.3 ± 1.3 min during exponential phase) while the Δ*ybeY(sup1)* strain grew at intermediate growth rate (doubling time of 36 ± 2.6 min during exponential phase) indicating partial suppression of the growth phenotype (Figure 1C). At 30°C, similar patterns of growth were observed (Figure 1D) with the doubling times during exponential phase for the *ybeY(*+), Δ*ybeY(sup1)* and Δ*ybeY::Kan* being 33.3 ± 2 min, 52.3 ± 4 min, and 82.2 ± 8.1 min, respectively. This result is consistent with our conclusion from the spotting assay that suppression in Δ*ybeY(sup1)* improves the growth independent of temperature. The suppression, however, is insufficient to improve temperature sensitivity at high temperatures.

Loss of *ybeY* causes severe ribosome assembly and 16S rRNA processing defects. To test the ribosome assembly status of Δ*ybeY(sup1)* strain, ribosomal particles from cell lysates were separated in sucrose gradients by ultracentrifugation to obtain the status of ribosome assembly. Compared to the *ybeY(*+) strain, Δ*ybeY::Kan* accumulates higher amounts of unassembled 30S and 50S subunits, with significantly reduced amounts of assembled 70S ribosomal particles (Figure 1E). The Δ*ybeY(sup1)* strain accumulated lower amounts of unassembled 30S and 50S subunits, with a modest corresponding increase in the levels of assembled 70S ribosomal particles. To test if rRNA processing defects are suppressed in the Δ*ybeY(sup1)*, total RNA was isolated and separated by gel electrophoresis. As expected, for the *ybeY(*+) strain, a single 16S rRNA band was formed but in contrast the Δ*ybeY::Kan* accumulates the previously reported larger 17S rRNA product and the shorter 16S* degradation product (Figure 1F). The total RNA from Δ*ybeY(sup1)* strain contained a reduced amount of 17S rRNA and markedly increased mature 16S rRNA indicating that the strain has improved 16S rRNA processing. Consistent with this inference, the strain did not accumulate significant amounts of 16S* degradation products. Taken together, these phenotypes suggested that the Δ*ybeY(sup1)* strain contained one or more suppressor mutations that improve growth, 16S rRNA processing, and modestly improve ribosome assembly.

### Whole Genome Sequencing Reveals the Presence of Suppressor Mutation in *era* Gene

**FIGURE 2:**
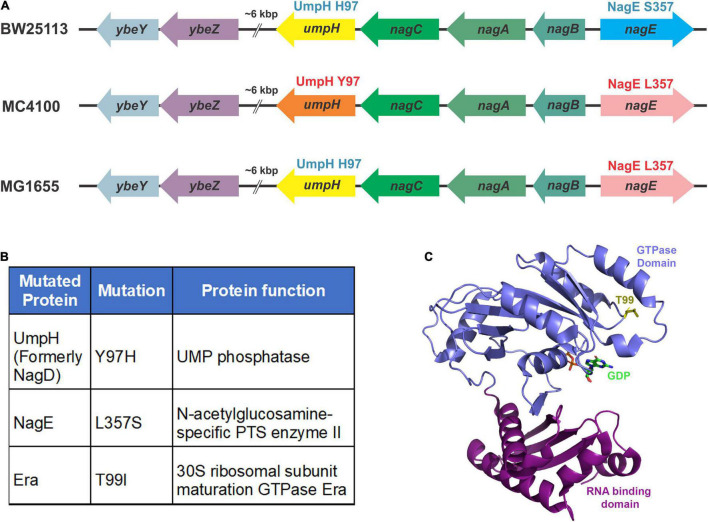
Mutations identified by whole-genome sequencing. **(A)** The variations in the *nagD* and *nagE* alleles in commonly used wild-type strains are indicated. The BW25113 strain carries the alleles that express UmpH H97 variant (represented by the yellow arrow) and NagE S357 variant (blue arrow). The MC4100 strain carries the alleles that express UmpH Y97 variant (orange arrow) and NagE L357 variant (pink arrow). The MG1655 strain carries the alleles that express UmpH H97 variant (yellow arrow) and NagE L357 variant (pink arrow). **(B)** Allelic differences between *ybeY(*+) and Δ*ybeY(sup1)* are indicated in the table as protein variants. **(C)** Location of the threonine 99 residue (yellow stick) in the GTPase domain (blue ribbon) indicated on the Era crystal structure (PDB: 3IEU). RNA binding domain is indicated as purple ribbon.

To identify the putative suppressor mutation(s), we performed a whole-genome sequencing analysis of the Δ*ybeY(sup1)* strain and as well *ybeY(*+) and Δ*ybeY::Kan* strains. Genomic differences between the strains were determined as described in section “Materials and Methods.” No differences were identified between the Δ*ybeY::Kan* strain and the *ybeY(*+) strain except for the *ybeY* deletion. However, we did note that the MC4100 background carries the UmpH Y97 and NagE L357 allelic variants, whereas strain BW25113, the parental strain of the Keio collection, carries the UmpH H97 and NagE S357 allelic variants (Figure 2A). The intensively studied strain MG1655 carries the UmpH H97 and NagE L357 combination. These allelic differences are important because the *umpH* and *nagE* genes are part of the *nag* gene cluster located at about 6000 base pairs away from the *ybeY* gene locus and are therefore genetically linked for P1 transduction. When we analyzed the Δ*ybeY(sup1)* strain, we found that it carried the UmpH H97 and NagE S357 from the Keio collection background, which indicated these allelic variants had indeed been co-transduced with the *ybeY* allele (Figure 2B). However, the Δ*ybeY(sup1)* strain also carried an unlinked mutation not found in the other strains, *era(T99I)*. Era is a highly conserved ribosome-associated GTPase composed of an N-terminal GTPase domain and a C-terminal RNA binding domain, and this T99I mutation alters the N-terminal GTPase domain of Era (Figure 2C). This led us to hypothesize that the suppression of Δ*ybeY* phenotypes was due to the *era(T99I)* mutation rather than the UmpH and NagE allele variants which vary between different *E. coli* lab strains. However, additional studies were required to test this hypothesis.

### *era(T99I)* Ameliorates Δ*ybeY* Growth Defect

To test whether the *era(T99I)* mutation alone contributes to the suppression of phenotypes in the Δ*ybeY(sup1)* strain without any contribution from the UmpH H97 and NagE S357 alleles, we first isolated and moved the mutation into the wild-type MC4100 strain to generate the *era(T99I) ybeY(*+) strain (see section “Materials and Methods” and Supplementary Figure 2). The *ybeY* gene was then replaced by the kanamycin resistance gene in the *era(T99I) ybeY(*+) strain to generate the *era(T99I)* Δ*ybeY* strain using the minimal step *ybeY* deletion method described earlier (Supplementary Figure 1). The strains were then subjected to whole-genome sequencing, which confirmed the absence of any additional mutations.

**FIGURE 3:**
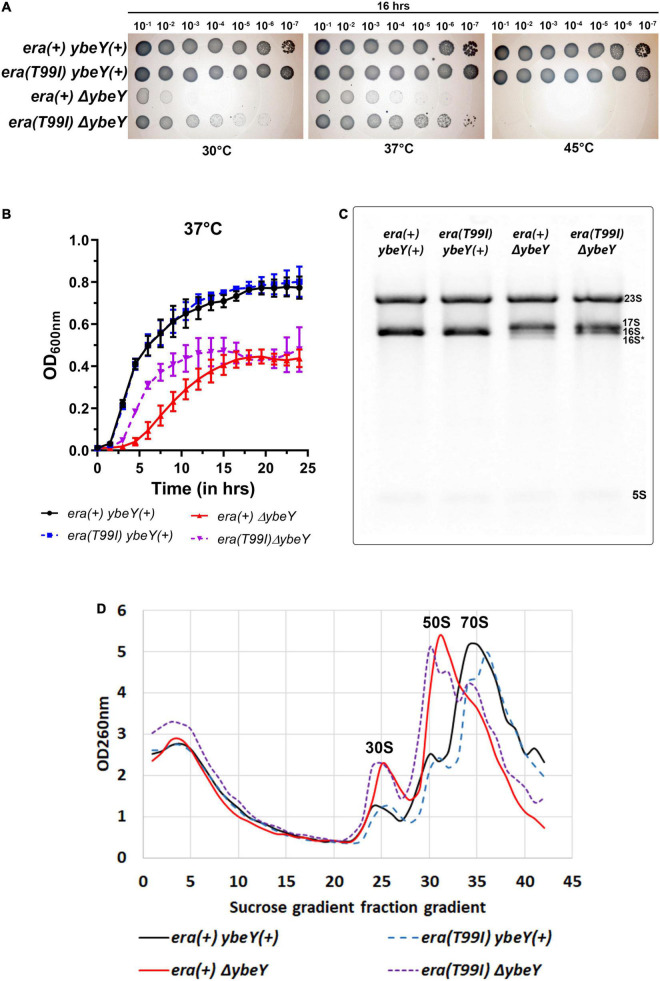
The *era(T99I)* mutation improves the growth defect of a Δ*ybeY*. **(A)** Tenfold serial dilutions of the overnight cultures were spotted onto LB agar plates and incubated at the indicated temperatures for 16 h. Representative sections of the imaged plates are shown. **(B)** Growth curves of strains *era(*+) *ybeY(*+) (black, continuous line, circles), *era(T99I) ybeY(*+) (blue, broken line, squares), *era(*+) Δ*ybeY* (red, continuous line, triangles), and *era(T99I)*Δ*ybeY* (purple, broken line, inverted triangles) determined in liquid LB media at 37°C by measuring OD at 600 nm in a plate reader for 24 h. The average ± SD of three determinations is shown. **(C)** Equal amounts of total RNA samples were separated on a Synergel-agarose gel by electrophoresis. A representative gel image of at least three independent determinations is shown. **(D)** Total cell lysate of the strains *era(*+) *ybeY(*+) (black, continuous line), *era(T99I) ybeY(*+) (blue, broken line), *era(*+) Δ*ybeY* (red, continuous line), and *era(T99I)*Δ*ybeY* (purple, broken line) separated by sucrose gradients and the amount of RNA was determined by measuring absorbance at 260 nm.

When we tested the effect of the *era(T99I)* on the ability of *ybeY(*+) and Δ*ybeY* strains to grow on solid LB agar medium at different temperatures, we obtained results that were extremely similar to those we observed for the Δ*ybeY(sup1)* strain (Figure 3A). To determine if the absence of growth after 16 h in both the *era(*+) and *era(T99I)*Δ*ybeY* strains is due to a slow-growth phenotype, the plates were then allowed to incubate at respective temperatures for an additional 24 h. All strains grew to confluence on the plates incubated at 30 and 37°C, however, no growth was observed for both the Δ*ybeY era(*+) and Δ*ybeY era(T99I)* at 45°C even after 40 h (Supplementary Figure 3).

We then determined the growth curves of the strains [*era(*+) *ybeY(*+), *era(T99I) ybeY(*+), *era(*+) Δ*ybeY*, and *era(T99I)* Δ*ybeY*] by inoculating LB media with overnight cultures. At 37°C, the wild type strains with and without *era(T99I)* grew similarly with the doubling times (average ± SD) at exponential phase for *era(*+) *ybeY(*+) and *era(T99I) ybeY(*+) strains being 29.02 ± 1.13 min and 29.69 ± 0.58 min, respectively (Figure 3B). On the other hand, in the strains lacking YbeY, the presence of the *era(T99I)* significantly improves the growth rate at 37°C with the doubling times at the exponential phase of *era(*+) Δ*ybeY* and *era(T99I)* Δ*ybeY* being 68.64 ± 6.02 min and 42.02 ± 0.43 min, respectively. A similar improvement in growth was observed at 30°C with the doubling times during the exponential phase for the *era(*+) *ybeY(*+), *era(T99I) ybeY(*+), *era(*+) Δ*ybeY*, and *era(T99I)* Δ*ybeY* being 39 ± 1.3 min, 39.7 ± 2.12 min, 132 ± 7.4 min, and 82.2 ± 5.1 min, respectively (Supplementary Figure 4). Taken together, these results indicate that the UmpH H97 and NagE S357 mutations are not relevant to the phenotypic suppression.

### *era(T99I)* Improves 16S rRNA Processing and Ribosome Assembly

We then tested if the *era(T99I)* mutation alone is responsible for the improvement in 16S rRNA processing in the Δ*ybeY(sup1)* strain. Compared to the *era(*+) Δ*ybeY* strain, the *era(T99I)*Δ*ybeY* strain has increased levels of the mature 16S rRNA band although it still accumulated significant amounts of immature 17S band (Figure 3C). We then tested if the *era(T99I)* mutation can ameliorate the reduction in the amount of 70S particles and accumulation of ribosomal subunits, 30S, and 50S seen in *era(*+) Δ*ybeY* strains. In a *ybeY(*+) background, the *era(T99I)* mutation had no effect on the relative amounts of mature 70S and precursor particles (30S and 50S) (Figure 3D). In contrast, in a Δ*ybeY* background the *era(T99I)* mutation substantially increased the ratio of mature 70S ribosomes to 30S and 50S ribosomal subunits, although not to the level seen in a wild type strain. These results indicate that the *era(T99I)* mutation is only partially able to suppress defects of a Δ*ybeY* at the ribosome level, similar to its ability to only partially suppress the growth phenotypes. However, these observations also raised the question as to whether the suppression is due to the increased stability of the 70S ribosome and/or 16S rRNA.

### *era(T99I)* Does Not Improve the Stability of 16S rRNA

**FIGURE 4:**
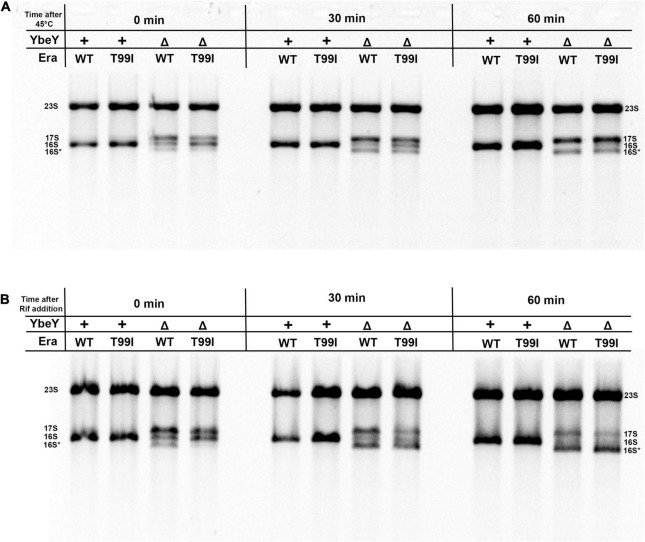
**(A)** Cell pellets were collected from the indicated strains grown to exponential phase at 37°C (0 min), after shifting to 45°C for 30 min and for 60 min. **(B)** Cell pellets were also collected from the indicated strains after adding 400 μM rifampicin for 30 min and 60 min at 37°C. Total RNA extracted from these samples was separated using Synergel-agarose gel electrophoresis. The disappearance of the 16S rRNA band over time is observed. Representative gel images of at least three independent determinations are shown.

We tested the stability of 16S rRNA under conditions of high temperature and also in the absence of transcription. The rRNA profiles of the strains under study were observed at different time intervals after shifting to high temperatures. In the *era(*+) Δ*ybeY* strain, the mature 16S rRNA levels decrease over time after the shift to 45°C from 37°C but the levels of 17S rRNA continue to persist (Figure 4A). This is likely because the processing of 17S rRNA into 16S rRNA halts and the existing 16S rRNA undergoes degradation. Hence, we conclude that YbeY is essential for proper 16S rRNA processing and stability at high temperatures. In the *era(T99I)*Δ*ybeY* strain, the levels of the different rRNA species eventually reach similar proportions to that of the *era(*+) Δ*ybeY* strain. This result indicates that the suppression due to *era(T99I)* completely disappears after the temperature shift and correlates well with the inability of the mutation to improve growth at 45°C. We then observed the rRNA profiles of the strains after the addition of the transcription inhibitor, rifampicin. In the *era(*+) Δ*ybeY* strain, both the amounts of 17S rRNA and the 16S rRNA species are reduced drastically with a corresponding increase in the intensity of the 16S* band (Figure 4B). This could be due to the processing of the existing 17S rRNA into 16S rRNA followed by degradation of 16S rRNA and/or direct misprocessing of 17S rRNA into 16S* species. Such degradation is not observed in *ybeY(*+) strains within the observed timeframe. This result supports the hypothesis that YbeY plays a role in the stability of the 16S rRNA and/or 17S rRNA. In the *era(T99I)*Δ*ybeY* strain, the decrease in 17S rRNA and 16S rRNA into the 16S* band appears to occur more rapidly compared to the *era(*+) Δ*ybeY* strain. These results suggest that *era(T99I)* mutation improves 16S rRNA processing but does not reduce its degradation.

### Effect of *era(T99I)* Mutation on Strains Lacking Exoribonucleases

Four exonucleases namely PNPase (*pnp*), RNase R (*rnr*), RNase II (*rnb*), and RNase PH (*rph*) have been implicated as acting redundantly in the 3′ end processing of the 16S rRNA (Sulthana and Deutscher, 2013). These exonucleases are also known to function in a multitude of other processes such as mRNA degradation, tRNA maturation, and sRNA maturation (Zuo and Deutscher, 2001; Li and Deutscher, 2004; Bechhofer and Deutscher, 2019). Our model for YbeY’s role in processing the 16S rRNA precursor (Jacob et al., 2013) proposes that, after the Era(GTP) has bound to the 3′ region of the 16S precursor to leave the terminal 33 nts protruding, YbeY acts endonucleolytically very close to the ultimate 16S 3′ terminus, leaving an 3′-phosphate end that is subsequently processed by one or more of the 3′ exoribonucleases. We subsequently suggested that the improved 16S processing we observed in a Δ*ybeY* mutant when Era is overproduced is due to exoribonucleases (RNase R, RNase PH, and RNase II, with a contribution from PNPase) acting together to remove the entire 33 precursor nucleotides from the 3′ terminus of 16S rRNA while it is bound to Era-GTP, with the extent of the processing being sterically limited by the Era protein (Ghosal et al., 2018).

**FIGURE 5:**
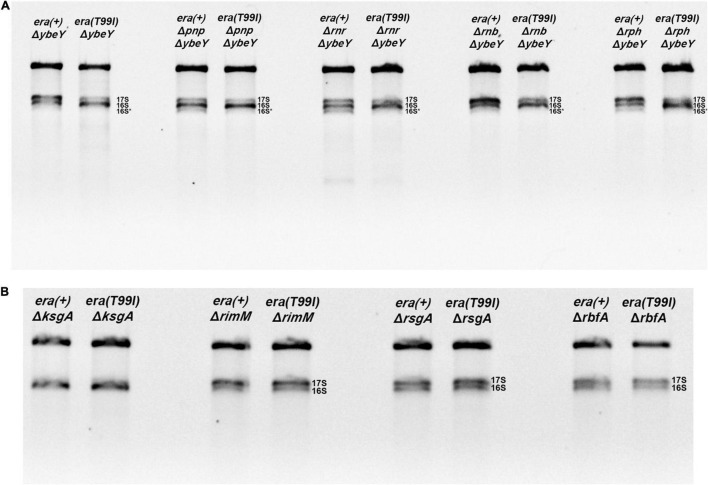
Total RNA extracted from the indicated strains were separated using Synergel-agarose gel electrophoresis. Representative gel images of at least three independent determinations is shown. **(A)** The Δ*ybeY* strains lacking one of the indicated exonucleases [PNPase (*pnp*), RNase R (*rnr*), RNase B (*rnb*), RNase PH (*rph*)] did not affect the *era(T99I)-*mediation suppression. The double mutants showed decrease in 17S rRNA and/or increase in 16S rRNA in the presence of *era(T99I)*. **(B)** The *era(T99I)* dependent improvement of 16S rRNA processing was not observed in the strains lacking one of the maturation factors (KsgA, RimM, RsgA, or RbfA).

To test whether the improved 16S processing we observed in Δ*ybeY era(T99I)* mutant resembled the improved processing we have previously described in a Δ*ybeY* mutant overexpressing Era, we introduced mutations inactivating PNPase, RNase R, RNase II, or RNase PH, into both *era(*+) and *era(T99I)* strains. As shown in Figure 5A, *era(T99I)* was able to improve the 16S rRNA processing in all the strains lacking one of the exonucleases as indicated by the decrease in 17S rRNA levels and/or increase in 16S rRNA levels. These results suggest that there is no absolute requirement for one of the exonucleases. These results differ from those we observed in the case of Era overexpression, where each mutation affecting a 3′ exoribonuclease diminished 16S processing somewhat (Ghosal et al., 2018). However, the redundancy of these exonucleases might allow one exonuclease to replace the other to play a role in the *era(T99I)* mediated suppression. In the course of these experiments, we noticed that the introduction of some of the exoribonuclease mutants into the Δ*ybeY* background further slowed the growth rate (Supplementary Figure 5), but these effects were observed regardless of whether the cells were *era(*+) and *era(T99I).* Nevertheless, in most cases, the presence of *era(T99I)* improved the growth of Δ*ybeY* cells regardless of the absence of one of the exoribonucleases.

### Effect of the *era(T99I)* Mutation on Strains Lacking Ribosome Maturation Factors

Next, we tested if the *era(T99I)* mutation can improve the defects caused by the absence of proteins that aid in ribosome assembly namely KsgA (RsmA; methyltransferase), RimM, RsgA (GTPase), and RbfA. Loss of KsgA has been shown by previous studies to confer mild cold sensitivity and result in the accumulation of 17S rRNA precursors at low temperatures (Connolly et al., 2008; Pletnev et al., 2020). These studies also showed that no significant amounts of unassembled ribosomal subunits were observed at 37°C and also only modest accumulation of 17S rRNA suggesting that KsgA plays a more important role in ribosome assembly at low temperatures. On the other hand, loss of one of the other maturation factors (RimM, RsgA, or RbfA) results in a significant amount of 17S rRNA accumulation (Bylund et al., 1998; Himeno et al., 2004). We made strains with combinations of *era(*+) or *era(T99I)* alleles together with the presence or absence of ribosome maturation factors. We examined the rRNA profile of the total RNA obtained from these strains grown to mid-exponential phase at 37°C. As expected, the strains lacking KsgA did not accumulate 17S rRNA precursors (Figure 5B). In contrast, the strains lacking either RsgA, RimM, or RbfA had significant amounts of 17S rRNA accumulation. Strikingly, the presence of the *era(T99I)* mutation did not improve the 16S rRNA processing in the strains lacking any of the ribosome maturation factors.

Additionally, in the strains lacking either KsgA or RsgA, the presence of *era(T99I)* mutation did not have any significant effect on growth at 37°C (Supplementary Figures 6A,B). On the other hand, strains lacking RimM or RbfA, namely *era(*+) Δ*rimM* and *era(*+) Δ*rbfA*, respectively, grew slowly. The *era(T99I)*Δ*rimM* and *era(T99I)*Δ*rbfA* double mutants grew similarly to their *era(*+) counterparts until the late exponential phase after which the strains with the *era(T99I)* exhibited an increased growth rate (Supplementary Figures 6C,D). This growth pattern is distinct from the suppression of Δ*ybeY* growth by *era(T99I)* where the improvement of growth consisted of an increase in growth rate throughout the exponential phase. Taken together, these results suggest that the *era(T99I)* mutation is a specific suppressor to loss of *ybeY* and not a general suppressor of ribosome assembly defects.

### *era(T99I)* Mutation Results in GroEL Accumulation in the 30S Fractions

**FIGURE 6:**
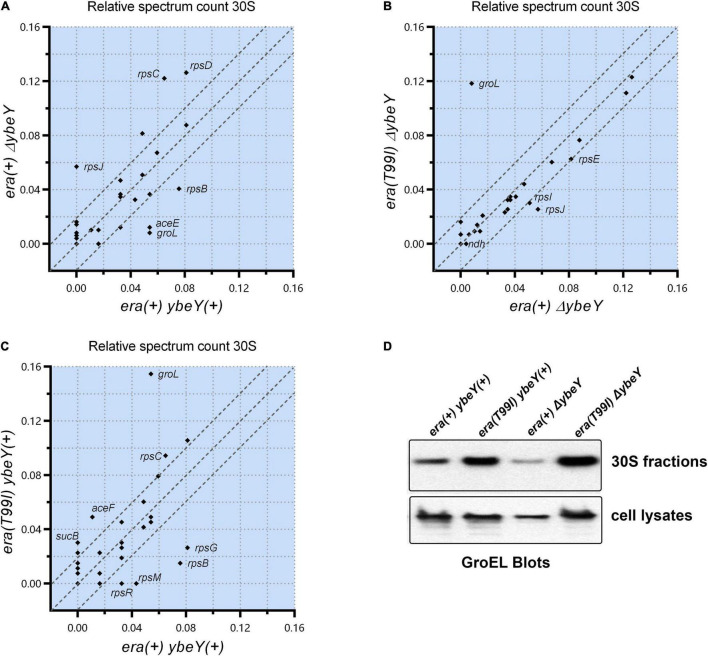
Relative spectrum count of the peptides identified by mass spectrometry is shown as comparison between strains *era(*+) *ybeY(*+) and *era(*+) Δ*ybeY* in **(A)**, *era(*+) Δ*ybeY* and *era(T99I)*Δ*ybeY* in **(B)**, and *era(*+) *ybeY(*+) and *era(T99I) ybeY(*+) in **(C)**. The data points are labeled by the gene name that expresses the identified peptide. The region between the parallel dotted lines represents the location of the peptides that differ by less than 20%. **(D)** Western blot analysis of GroEL protein levels in the 30S fractions (upper panel) and in the cell lysates (lower panel) from the indicated strains.

To determine if the *era(T99I)* mutation alters the protein composition of the ribosomal particles, we performed mass spectrometry analysis of the 30S, 50S, and 70S subunit fractions from the strains under study and compared the spectrum counts. There was a significant difference between the *era(*+) *ybeY(*+) and *era(*+) Δ*ybeY* strains in the amounts of certain proteins in the 30S ribosomal fractions. Specifically, *era(*+) Δ*ybeY* 30S fractions contained more detectable S3 (*rpsC*), S4 (*rpsD*), and S10 (*rpsJ*) compared to *era(*+) *ybeY(*+) (Figure 6A). In contrast, S2 (*rpsB*) amounts were lower in Δ*ybeY era(*+) compared to *ybeY(*+) *era(*+). This result shows that the absence of YbeY causes aberrant 30S subunit assembly. To understand the effect of the *era(T99I)* in this process, we compared the amounts of proteins in the 30S ribosomal fractions between the Δ*ybeY era(*+) strain and Δ*ybeY era(T99I)*. Surprisingly, there was little difference in the 30S protein composition between the two strains except for the significant amount of GroEL (a component of the GroEL-GroES chaperonin) detected in the *era(T99I)* Δ*ybeY* (Figure 6B). We then compared *era(*+) *ybeY(*+) strain and *era(T99I) ybeY(*+) to determine if GroEL accumulation in the 30S fractions is dependent on *era(T99I)* alone. As shown in Figure 6C, the *era(T99I) ybeY(*+) strain also accumulated GroEL similar to the *era(T99I)*Δ*ybeY* strain. This result indicates that GroEL accumulation in 30S fractions is due to the *era(T99I)* mutation and is independent of the presence or absence of *ybeY*.

Similar analysis of 50S and 70S subunits revealed no significant difference in the protein composition between the *era(*+) Δ*ybeY* strain and the *era(T99I)* Δ*ybeY* (Supplementary Figure 7). Using Western blotting, we then tested if the increase in GroEL levels in the 30S fractions due to *era(T99I)* is due to an increase in protein levels of GroEL. Whole cell lysate blots reveal no significant increase in GroEL levels in the strains carrying *era(T99I)* compared to the *era(*+) *ybeY(*+) strain (Figure 6D). In contrast, the 30S fraction blots confirm the mass spectrometry finding that the GroEL levels are higher in strains carrying *era(T99I)*. This result suggests that the *era(T99I)* mutation is responsible for the accumulation of GroEL in 30S fractions but not via altering the protein levels of GroEL.

### Overexpression of the GroEL-GroES Chaperonin Is Insufficient for Suppression of Δ*ybeY* Phenotypes

**FIGURE 7:**
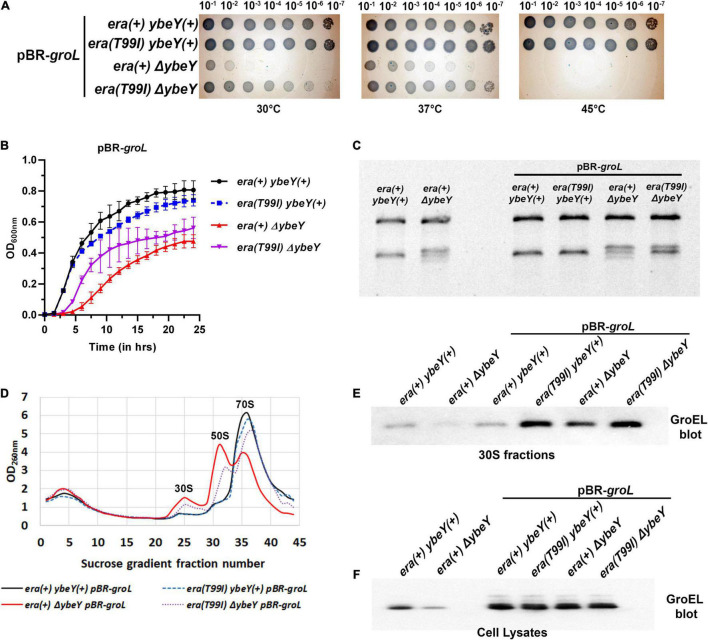
**(A)** Tenfold serial dilutions of the overnight cultures of the indicated strains were spotted onto LB agar plates and incubated at the indicated temperatures for 16 h. Representative sections of the imaged plates are shown. **(B)** Growth curves of strains *era(*+) *ybeY(*+) pBR-*groL* (black, continuous line, circles), *era(T99I) ybeY(*+) pBR-*groL* (blue, broken line, squares), *era(*+) Δ*ybeY* pBR-*groL* (red, continuous line, triangles), and *era(T99I)*Δ*ybeY* pBR-*groL* (purple, broken line, inverted triangles) determined in liquid LB media at 37°C by measuring OD at 600 nm in a plate reader for 24 h. The average ± SD of three determinations is shown. **(C)** Total RNA extracted from the indicated strains was separated using Synergel-agarose gel electrophoresis. Representative gel images of at least three independent determinations is shown. **(D)** Total cell lysate of the strains *era(*+) *ybeY(*+) pBR-*groL* (black, continuous line), *era(T99I) ybeY(*+) pBR-*groL* (blue, broken line), *era(*+) Δ*ybeY* pBR-*groL* (red, continuous line), and *era(T99I)*Δ*ybeY* pBR-*groL* (purple, broken line) separated by sucrose gradients and the amount of RNA was determined by measuring absorbance at 260 nm. Western blot analysis of GroEL protein levels in the 30S fractions **(E)** and in the cell lysates **(F)** from the indicated strains.

The finding that GroEL accumulates in the *era(T99I)* strains raises an additional question of whether overexpression of the GroEL-GroES chaperonin can increase GroEL levels in 30S fractions and suppress Δ*ybeY* phenotypes. To that end, we generated strains expressing GroEL and GroES from a multi-copy plasmid (*pBR-groL*). First, in a spotting assay, both the empty vector pBR322 and pBR-*groL* did not improve the growth of the *era(*+) Δ*ybeY* strain at both 30 and 37°C, and did not suppress the heat sensitivity phenotype at 45°C (Supplementary Figure 8 and Figure 7A). Similarly, in liquid culture growth curves at 37°C, pBR-*groL* did not confer growth improvement of the Δ*ybeY* strain (Figure 7B). Also, it is to be noted that the overexpression of the GroEL-GroES chaperonin did not interfere with the growth improvement due to *era(T99I)*. Next, rRNA profile of the *era(*+) Δ*ybeY* pBR-*groL* was indistinguishable from that of the *era(*+) Δ*ybeY* strain that does not overproduce *groL* (Figure 7C). Furthermore, sucrose gradient analysis of ribosome subunits revealed no suppression of the ribosome assembly defect by overexpression of *groL* (Figure 7D). Similar to the growth phenotypes, pBR-*groL* had no effect on the *era(T99I)* mediated suppression of the 16S rRNA processing and ribosome assembly defects. Western blot analysis of the 30S fractions of the strains expressing pBR-*groL* exhibited increase in *groEL* accumulation in strains expressing *era(T99I)*, namely, *ybeY(*+) *era(T99I) pBR-groL and*Δ*ybeY era(T99I) pBR-groL* (Figure 7E). Interestingly, Δ*ybeY era(*+) *pBR-groL* strain also showed increase in GroEL levels compared to Δ*ybeY era(*+) although the accumulation was not as striking as in *era(T99I)* strains. However, this increase in GroEL levels did not result in the suppression of Δ*ybeY* phenotypes. No such increase was observed for the *ybeY(*+) *era(*+) pBR-*groL* strain. This was despite all strains with pBR-*groL* contained equally high levels of GroEL as determined by the whole cell lysate blots (Figure 7F). Taken together, these results indicate that overexpression of the GroEL-GroES chaperonin is not sufficient to mimic *era(T99I)* mediated suppression.

## Discussion

The slow growth phenotype of *ybeY* deletion strains allowed us to identify suppressed strains that formed relatively large colonies. Identification of the suppressor in the *era* gene in one such suppressed strain supports the model where YbeY, Era, and S11 act together in ribosome assembly and 16S rRNA processing (Vercruysse et al., 2016; Ghosal et al., 2018). The crystal structure of Era (Tu et al., 2009) indicates that Era protein binds near the 3′ end of the 16S rRNA. Era interactions with S11 and YbeY position these three proteins at the 3′ end suggesting a model where these proteins together facilitate 16S rRNA processing and ribosome assembly. In human mitochondria, this three-way interaction has been suggested to play an important role in ribosome assembly although not in rRNA processing. Nevertheless, the Era-S11-YbeY interactions seem to be fundamentally important and therefore highly conserved from bacteria to humans. In the absence of YbeY protein, acquisition of suppressor mutation in Era might allow for stabilization of the interaction with S11 and/or other proteins required for 16S rRNA processing and ribosome assembly.

**FIGURE 8:**
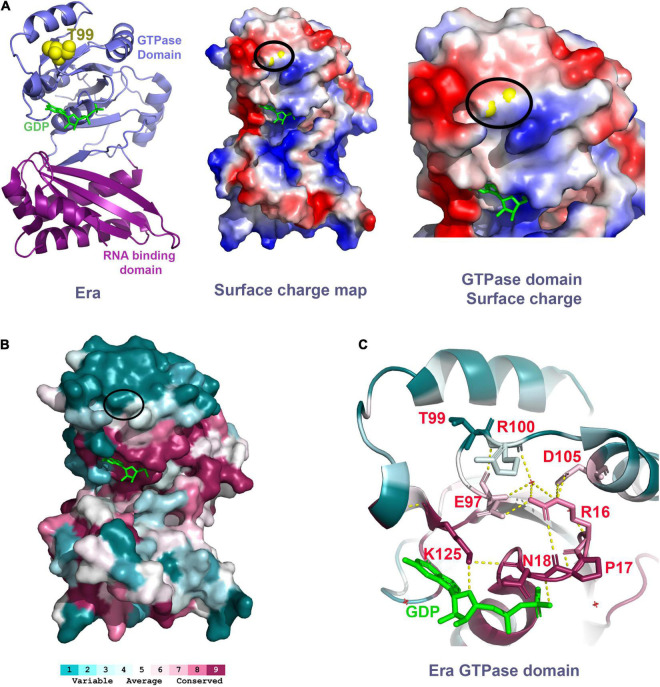
**(A)** Left panel shows the Era structure (PDB: 3IEU) with T99 residue (yellow spheres) and GDP (green). Middle panel shows the charge distribution of the Era protein at the surface that has the T99 residue (yellow spheres within the black oval). Right panel shows the close-up view of the GTPase domain with the T99 residue (yellow spheres within the black oval). Negative charge – Red. Positive charge – Blue. **(B)** Era protein surface colored based on the conservation scores obtained using ConSurf tool. Black oval highlights the location of the T99 residue. **(C)** Polar interactions between the residues highlighted (sticks, red labels) are indicated as the dashed yellow lines.

Threonine-99 residue of the Era protein is located in the loop between beta-sheet 5 (β5) and alpha-helix 3 (α3) of the GTPase domain. The phenotypic suppression occurs due to the amino acid change from a polar residue threonine to isoleucine, a hydrophobic residue. This threonine residue is surface exposed in the crystal structure of Era (PDB: 3IEU), which will be disrupted by the introduction of the hydrophobic residue. Therefore, we predict that this single residue change might cause local structural changes. Secondary structure analysis of the Era(T99I) variant using SWISS-MODEL (Waterhouse et al., 2018) suggests a minor structural change at the loop with Isoleucine 99 (Supplementary Figure 9). T99 residue is located at the surface of the Era protein on the same side of the GTPase domain that contains the GDP/GTP binding pocket. The surface charge map shows that this uncharged polar residue is located between a positively charged surface region and a negatively charged surface region (Figure 8A). The threonine to Isoleucine residue change introduces hydrophobicity to the region that might be involved in protein-protein interactions.

To further understand the importance of the T99 residue and its interacting residues, we performed a residue conservation analysis using the ConSurf tool (Ashkenazy et al., 2016). The threonine-99 is not an evolutionarily conserved residue. It is located within a cluster of less conserved amino acid residues at the upper surface of the GTPase domain away from the RNA binding domain (Figure 8B). However, we found that the T99 residue is closer to a conserved E97 residue located at parallelly opposite turn within the same loop as T99. The E97 residue makes critical polar interactions with the highly conserved residue R16 which is part of a most conserved loop (R16, P17, and N18) that coordinates the GDP/GTP binding (Figure 8C). The E97 residue also interacts with the R100 residue, the residue that follows T99. The structural change due to the mutation of T99 residue to Isoleucine could alter the interactions of E97 and R100 thereby indirectly affecting the GDP/GTP binding to Era and/or its GTPase activity. Since the T99 residue is not directly involved in GTP/GDP binding, mutating it causes modest effects and avoids the possible drastic effects if a highly conserved residue that is directly involved in GTP/GDP was mutated. The cryo-EM structure of Era bound to 30S ribosomal subunit also showed that the GTPase domain of Era can also bind to helix 26 of the 16S rRNA and also to the S18 protein (Sharma et al., 2005). Although these interactions are primarily close to the NTD and CTD linker region, mutation in the GTPase domain could have some effect.

Era and its binding partners function both to hold the 16S rRNA precursor for proper processing and as also a checkpoint for ribosome assembly to proceed. GTPase activity is stimulated by its binding to 16S rRNA (Tu et al., 2011). Four exonucleases can redundantly function to process the 3′ end of the 16S rRNA and are likely responsible for the correctly processed 16S that is observed in a Δ*ybeY* mutant (Sulthana and Deutscher, 2013). The presence of Era(T99I) mutation could improve Era binding and extend its half-life on the 16S rRNA precursor thereby giving RNase R, RNase II, RNase PH, and PNPase additional time to compensate for the loss of YbeY protein allowing the exonucleases by performing exoribonucleolytic processing. However, this alternative 16S processing system is inefficient relatively compared to the normal processing events that happen in the presence of YbeY, as indicated by the partial suppression of phenotypes.

The GroEL chaperone is a large complex that sediments close to 30S fractions. The identification of GroEL in our samples could be a result of some carry-over. However, the accumulation of GroEL was particularly higher in cells with Era(T99I). This might be due to its interaction with Era(T99I) variant causing increase in the size of the complex pushing it further to sediment with the 30S fraction. Another possibility is that the GroEL complex could be recruited to the 30S subunit by Era(T99I). The recruitment model of GroEL-GroES chaperone to the 30S subunit in the presence of the Era(T99I) is interesting in the 16S processing context as it may also play a role in stabilizing the Era complex to allow for proper processing. However, this recruitment also happens in the presence of YbeY and overexpression of GroEL-GroES alone did not result in the accumulation of GroEL in the 30S subunit. This suggests that GroEL-GroES could be functioning as a direct chaperone for the Era(T99I) protein variant.

One formal possibility for how threonine-99 might play a role in Era function is by post-translational modifications. Specifically, threonine residues are shown to be one of the amino acids that get phosphorylated in bacteria similar to eukaryotes (Macek et al., 2008). However, the bacterial phosphorylation prediction tools, namely NetPhosBac (Miller et al., 2009) and cPhosBac (Li et al., 2015), did not predict that the threonine-99 residue as a phosphorylation site. Moreover, although threonine-99 is on the protein surface, the Era structure suggests that it would not be easily accessible to a kinase. The absence of conservation of this residue also suggests that the probability of T99 residue to undergo functionally important post-translational modifications is low. Our previous study showed that overexpression of Era suppresses phenotypes due to loss of YbeY (Ghosal et al., 2018). This leads to another possibility that the presence of T99I mutation might increase the expression level of Era leading to the suppression. However, expression levels of era were only modestly higher in *era(T99I)* compared to the *era*+ in the *ybeY*+ background (Supplementary Figure 10). Moreover, in the Δ*ybeY* background, expression levels of *era* were not significantly different between *era*+ and *era(T99I)*.

In this study, we also highlight two conditions where YbeY is absolutely essential for 16S rRNA processing and stability. Absence of YbeY results in an accumulation of 16S* band formed by the degradation of the 16S rRNA. The *era(T99I)* mutation could not rescue the degradation of the 16S rRNA over time when the transcription is halted by Rifampicin. This result suggests the indispensable role played by YbeY in 16S rRNA stability and cells lacking YbeY require continuous transcription of ribosomal RNA to compensate for the degradation. Similarly, the absence of YbeY results in an accumulation of 17S band at 45°C. YbeY is a heat shock protein and its expression is modulated by the heat shock sigma factor (σ32) (Rasouly et al., 2009). The *era(T99I)* mutation could not improve the conversion of 17S rRNA into 16S rRNA highlighting the requirement for YbeY at high temperatures for 16S rRNA processing.

## Data Availability Statement

The data presented in the study are deposited in the NCBI Bioproject repository (http://www.ncbi.nlm. nih.gov/bioproject/814708), accession number: PRJNA814708.

## Author Contributions

VB and GW designed the research and experiments, analyzed the data, made the figures, and wrote the manuscript. VB, SS, and AG performed the experiments. All authors contributed to the article and approved the submitted version.

## Conflict of Interest

The authors declare that the research was conducted in the absence of any commercial or financial relationships that could be construed as a potential conflict of interest.

## Publisher’s Note

All claims expressed in this article are solely those of the authors and do not necessarily represent those of their affiliated organizations, or those of the publisher, the editors and the reviewers. Any product that may be evaluated in this article, or claim that may be made by its manufacturer, is not guaranteed or endorsed by the publisher.

## Footnotes

1
https://usegalaxy.org/
