# Supplementary material for: A Mutant Era GTPase Suppresses Phenotypes Caused by Loss of Highly Conserved YbeY Protein in Escherichia coli
Source: Front Microbiol. 2022 May 19;13:896075. doi: 10.3389/fmicb.2022.896075 (PMC9159920; doi:10.3389/fmicb.2022.896075)
Supplement: Supplementary file 1 [file Presentation_1.pdf]

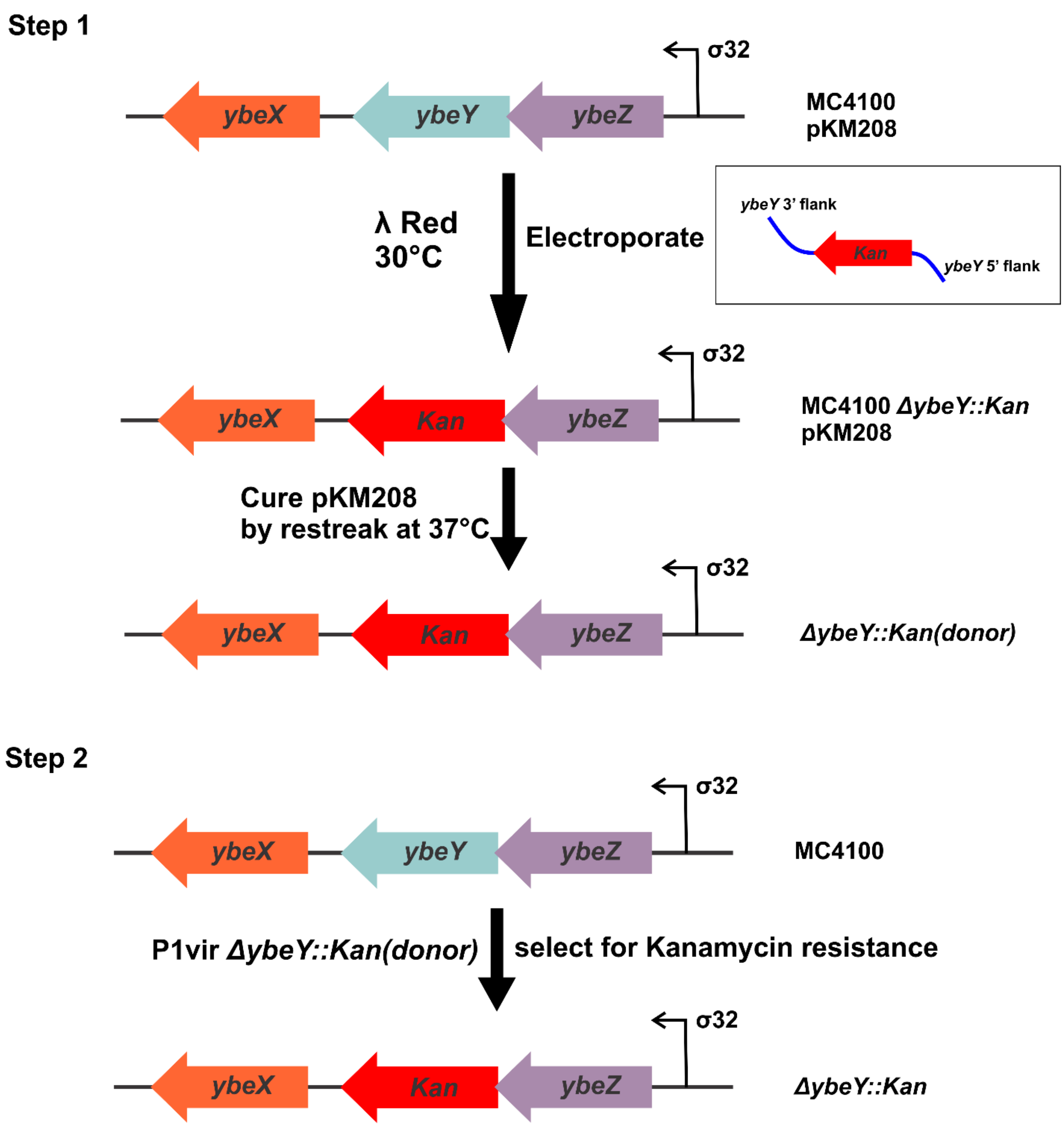

Supplementary Figure 1. Schematic representation of the steps involved in generating *ΔybeY::Kan* strain avoiding linked mutations in Keio collection.

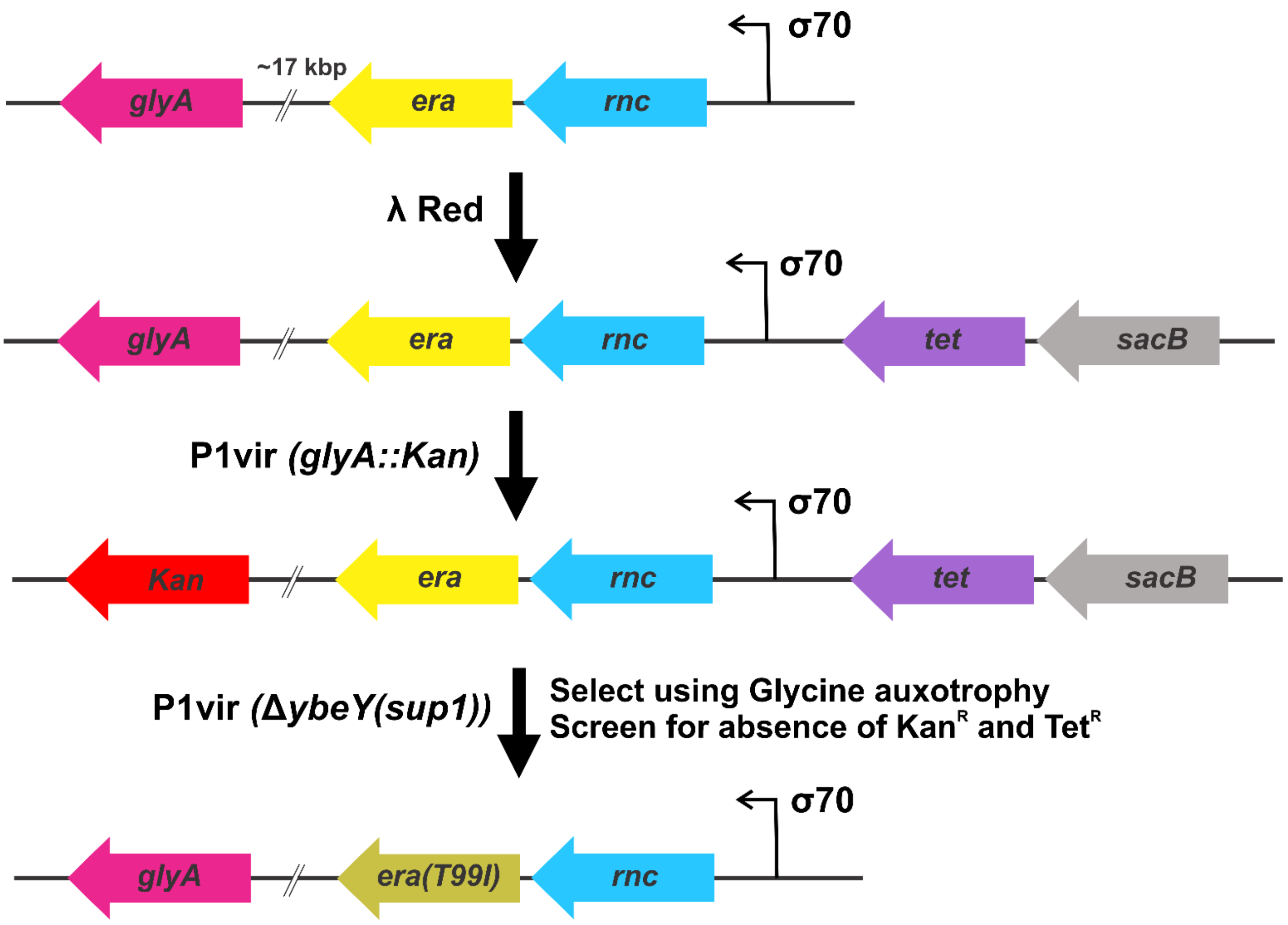

Supplementary Figure 2. Schematic representation of the steps involved in moving *era(T99I)* mutation from *ΔybeY(sup1)* strain into wild type MC4100 (*ybeY(+)*) to generate the *era(T99I) ybeY(+)* strain.

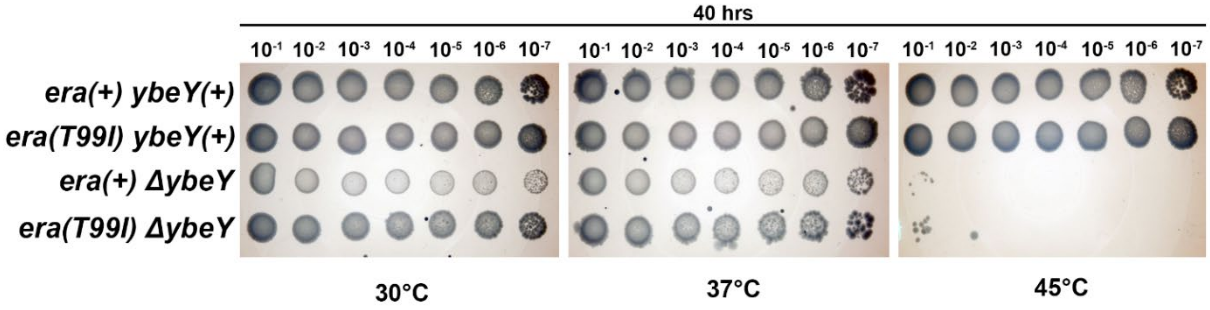

Supplementary Figure 3. Spotting assay shown in Figure 3C was allowed to incubate at respective temperature for additional 24 hours for a total of 40 hours.

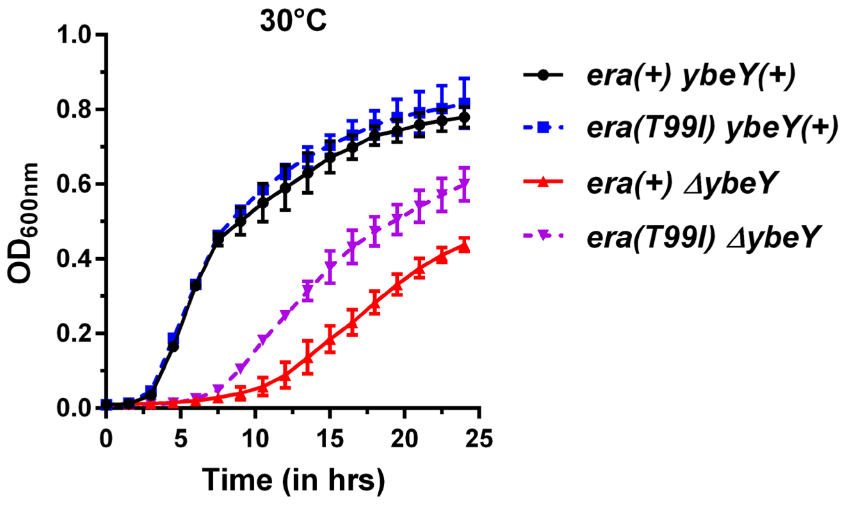

Supplementary Figure 4. The *era(T99I)* mutation improves the growth defect due to loss of YbeY at 30°C in liquid LB media.

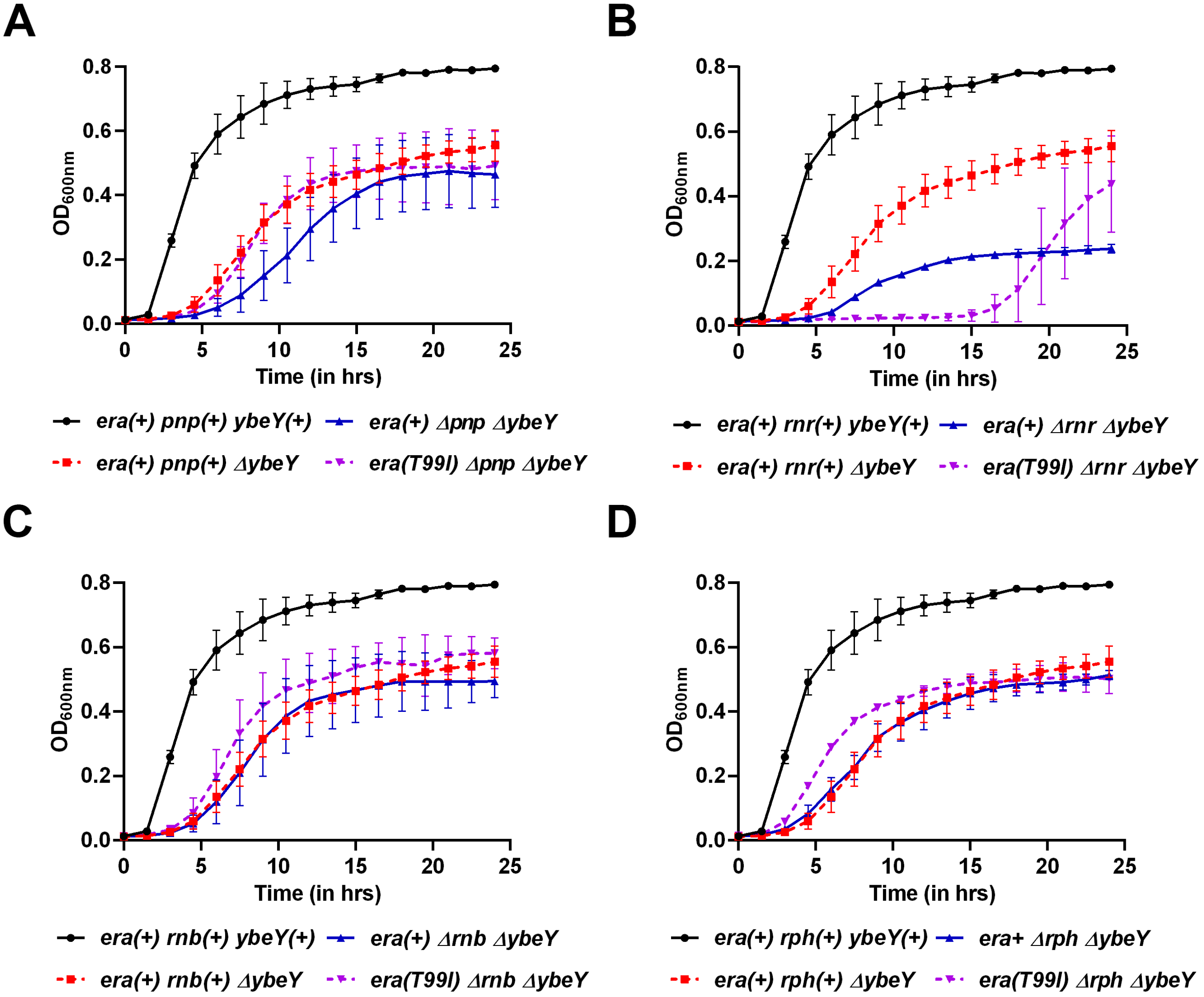

Supplementary Figure 5. **| (A)** Growth curves of strains *era(+) Δpnp ΔybeY* (blue, continuous line, triangles), and *era(T99I) Δpnp ΔybeY* (purple, broken line, inverted triangles). **(B)** Growth curves of strains *era(+) Δrnr ΔybeY* (blue, continuous line, triangles), and *era(T99I) Δrnr ΔybeY* (purple, broken line, inverted triangles). **(C)** Growth curves of strains *era(+) Δrnb ΔybeY* (blue, continuous line, triangles), and *era(T99I) Δrnb ΔybeY* (purple, broken line, inverted triangles). **(D)** Growth curves of strains *era(+) Δrph ΔybeY* (blue, continuous line, triangles) and *era(T99I) Δrph ΔybeY* (purple, broken line, inverted triangles). Wild type strain [*era(+) pnp(+) rnr(+) rnb(+) rph(+) ybeY(+)*] (black, continuous line, circles) and YbeY deletion strain [*era(+) pnp(+) rnr(+) rnb(+) rph(+) ΔybeY*] (red, broken line, squares) are shown in all graphs for comparison. Growth curves were determined in liquid LB media at 37°C by measuring OD at 600 nm in a plate reader for 24 hrs. The average ± SD of three determinations is shown.

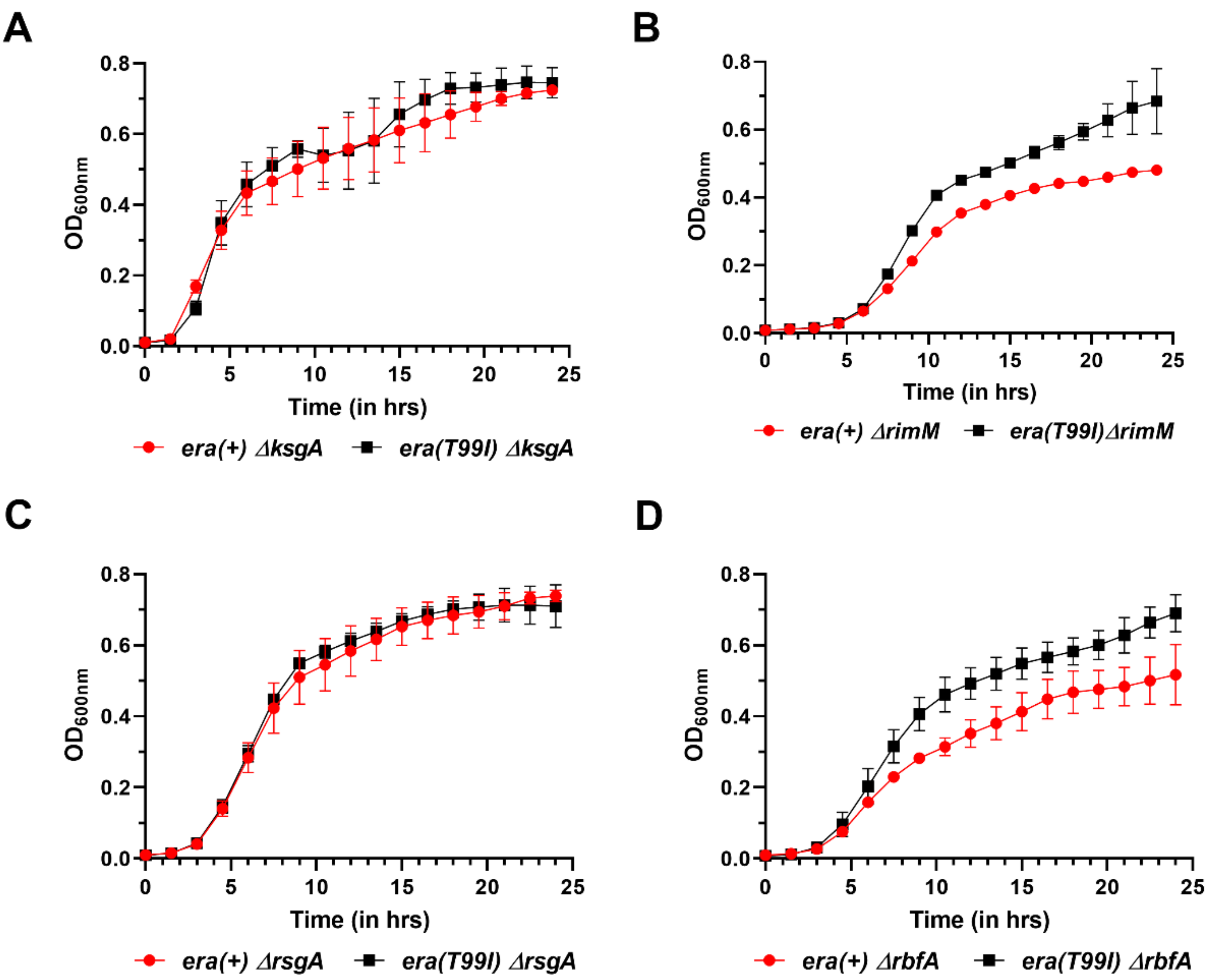

Supplementary Figure 6. Growth curves of strains, **(A)** *era(+) ΔksgA* (red, circles, doubling time (DT): 30.03 ± 1.66 min) and *era(T99I) ΔksgA* (black, squares, DT: 31.94 ± 0.77 min), **(B)** *era(+) ΔrimM* (red, circles, DT: 76.88 ± 5.62 min) and *era(T99I) ΔrimM* (black, squares, DT: 67.70 ± 4.41 min)**, (C)** *era(+) ΔrsgA* (red, circles, DT: 49.21 ± 4.58 min) and *era(T99I) ΔrsgA* (black, squares, DT: 47.3269 ± 0.45 min)**, (D)** *era(+) ΔrbfA* (red, circles, DT: 59.98 ± 1.17 min) and *era(T99I) ΔrbfA* (black, squares, DT: 55.06 ± 6.52 min) determined in liquid LB media at 37°C by measuring OD at 600 nm in a plate reader for 24 hrs. The average ± SD of three determinations is shown.

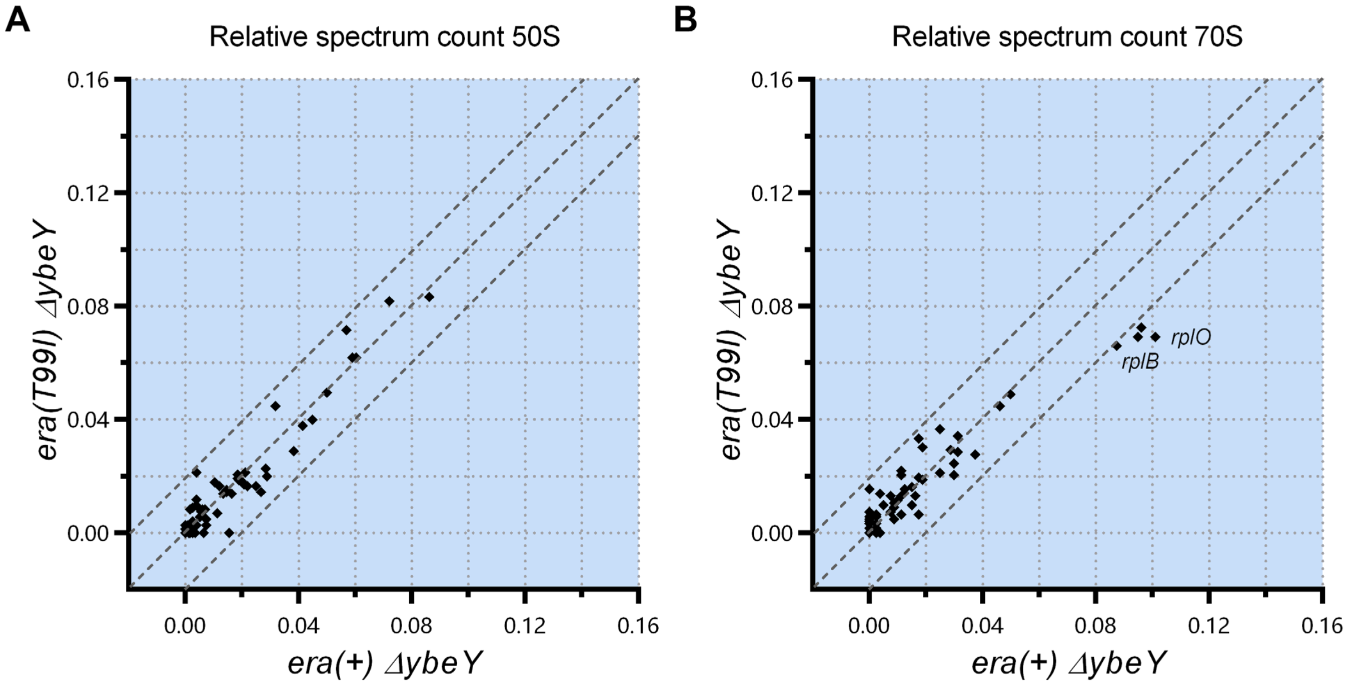

Supplementary Figure 7. Relative spectrum count of the 50S **(A)** and 70S **(B)** samples determined by mass spectrometry represented as a comparison between *ΔybeY era(+)* and *era(T99I) ΔybeY.*

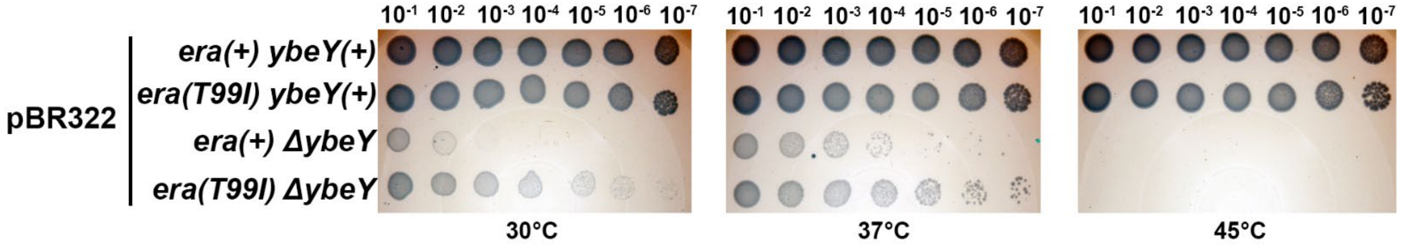

Supplementary Figure 8. Ten-fold serial dilutions of the overnight cultures of the indicated strains were spotted onto LB agar plates and incubated at the indicated temperatures for 16hrs. Representative sections of the imaged plates are shown. Presence of pBR322 empty vector has no significant effects on the growth pattern of the strains when compared to Figure 3B.

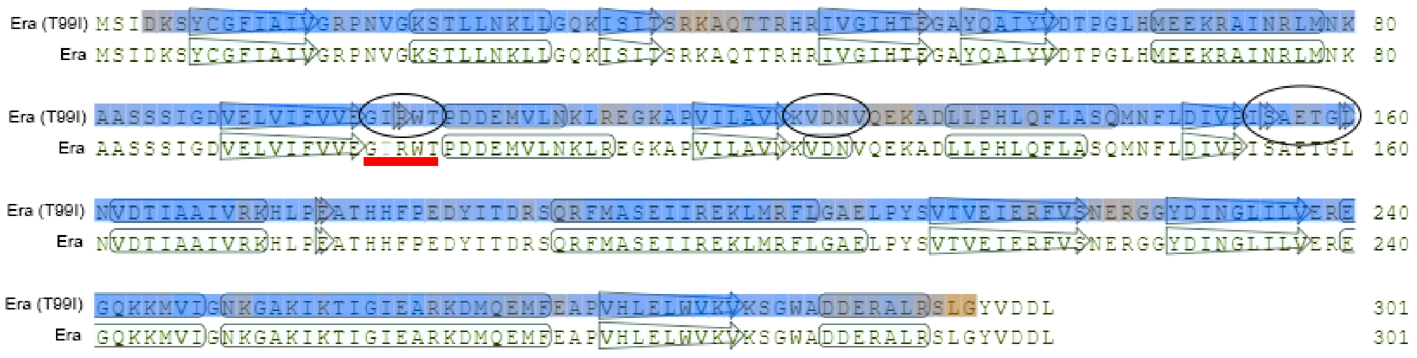

Supplementary Figure 9. Secondary structure prediction of Era (T99I) variant sequence aligned with wild type Era sequence. Red line highlights the loop that has the T99 residue. Arrow outline - β sheet. Rectangle outline – α helix. Black ellipses highlight the possible minor structural changes in Era (T99I).

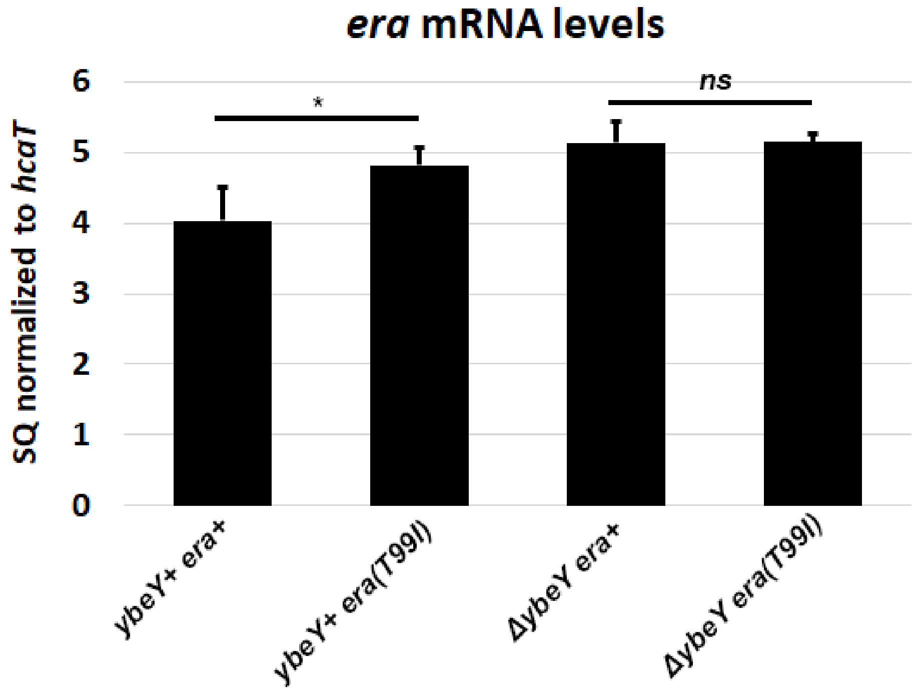

Supplementary Figure 10. Expression levels of *era* gene in the indicated strains as determined by qPCR. The starting quantities (SQ) were normalized to the expression levels of the control *hcaT* gene. One Way ANOVA was used to determine the statistical significance of the difference in expression levels. * P<0.01; ns - not significant.

Supplementary references

Cherepanov, P. P., and Wackernagel, W. (1995). Gene disruption in Escherichia coli: TcR and KmR cassettes with the option of Flp-catalyzed excision of the antibiotic-resistance determinant. *Gene* 158, 9–14. doi:10.1016/0378-1119(95)00193-A.

Li, X. T., Thomason, L. C., Sawitzke, J. A., Costantino, N., and Court, D. L. (2013). Positive and negative selection using the tetA-sacB cassette: Recombineering and P1 transduction in Escherichia coli. *Nucleic Acids Res.* 41. doi:10.1093/nar/gkt1075.

Murphy, K. C., and Campellone, K. G. (2003). Lambda Red-mediated recombinogenic engineering of enterohemorrhagic and enteropathogenic E. coli. *BMC Mol. Biol.* 4. doi:10.1186/1471-2199-4-11.
