# Supplementary material for: A Mutant Era GTPase Suppresses Phenotypes Caused by Loss of Highly Conserved YbeY Protein in Escherichia coli
Source: Front Microbiol. 2022 May 19;13:896075. doi: 10.3389/fmicb.2022.896075 (PMC9159920; doi:10.3389/fmicb.2022.896075)
Supplement: Supplementary file 2 [file Table_1.DOCX]

**Table. Strains, plasmids and primers used in this study**

| ***Escherichia coli* strains** | | |
| --- | --- | --- |
| Strain name | Relevant characteristics | Source |
| MC4100 | Wild type *ybeY+* | Laboratory stock |
| JW0656 | BW25113 *ΔybeY* | Keio collection |
| *ΔybeY(sup1)* | Suppressed *ΔybeY* isolate 1 | This work |
| *ΔybeY::Kan* | MC4100 *ΔybeY* (Kan^R^) | This work |
| XTL298 | Source of *tetA-sacB* cassette | (Li et al., 2013) |
| VB030 | *era(T99I) ybeY(+)* | This work |
| VB031 | *era(T99I) ΔybeY::Kan* | This work |
| VB032 | *era+ ΔksgA::Kan* | This work |
| VB033 | *era(T99I) ΔksgA::Kan* | This work |
| VB034 | *era+ ΔrimM::Kan* | This work |
| VB035 | *era(T99I) ΔrimM::Kan* | This work |
| VB036 | *era+ ΔrsgA::Kan* | This work |
| VB037 | *era(T99I) ΔrsgA::Kan* | This work |
| VB038 | *era+ ΔrbfA::Kan* | This work |
| VB039 | *era(T99I) ΔrbfA::Kan* | This work |
| VB040 | *era+ Δpnp::Kan ΔybeY::cam* | This work |
| VB041 | *era(T99I) Δpnp::Kan ΔybeY::cam* | This work |
| VB042 | *era+ Δrnr::Kan ΔybeY::cam* | This work |
| VB043 | *era(T99I) Δrnr::Kan ΔybeY::cam* | This work |
| VB044 | *era+ Δrnb::Kan ΔybeY::cam* | This work |
| VB045 | *era(T99I) Δrnb::Kan ΔybeY::cam* | This work |
| VB046 | *era+ Δrph::Kan ΔybeY::cam* | This work |
| VB047 | *era(T99I) Δrph::Kan ΔybeY::cam* | This work |
| **Plasmids** | | |
| Plasmid name | Relevant characteristics | Source and Reference |
| pCP20 | Flp recombinase | CGSC; (Cherepanov and Wackernagel, 1995) |
| pKM208 | P*tac-red-gam;* Origin Ts | Addgene; (Murphy and Campellone, 2003) |
| pBR-*groL* | pBR322 plasmid expressing GroEL | This work |
| **Primers** | | |
| Primer Name | Sequence | |
| rncuptetAFor | cgaatggccgactggtctgcgcttaagtcgcattggcggcatccattaattcctaatttttgttgacactctatc | |
| rncupSacBRev | ccacgggagatttatctcataaataattcacgttgtcgccataacggcgaatcaaagggaaaactgtccatatg | |
| FP_ybeY_KO | gcggcgctggcagcagaacgcaagcgcgaagaacaggaacaaaaatgagtgtgtaggctggagctgcttc | |
| RP_ybeY_KO | gttaatcaccaacggcggggacgtctgccagtcaaatgcctggcaaattacatatgaatatcctccttag | |
